# Leucocyte Telomere Length and Risk of Type 2 Diabetes Mellitus: New Prospective Cohort Study and Literature-Based Meta-Analysis

**DOI:** 10.1371/journal.pone.0112483

**Published:** 2014-11-12

**Authors:** Peter Willeit, Julia Raschenberger, Emma E. Heydon, Sotirios Tsimikas, Margot Haun, Agnes Mayr, Siegfried Weger, Joseph L. Witztum, Adam S. Butterworth, Johann Willeit, Florian Kronenberg, Stefan Kiechl

**Affiliations:** 1 Department of Neurology, Innsbruck Medical University, Innsbruck, Austria; 2 Department of Public Health and Primary Care, University of Cambridge, Cambridge, United Kingdom; 3 Division of Genetic Epidemiology, Innsbruck Medical University, Innsbruck, Austria; 4 Department of Medicine, University of California San Diego, La Jolla, United States of America; 5 Department of Laboratory Medicine, Bruneck Hospital, Bruneck, Italy; 6 Department of Internal Medicine, Bruneck Hospital, Bruneck, Italy; Tulane University Health Sciences Center, United States of America

## Abstract

**Background:**

Short telomeres have been linked to various age-related diseases. We aimed to assess the association of telomere length with incident type 2 diabetes mellitus (T2DM) in prospective cohort studies.

**Methods:**

Leucocyte relative telomere length (RTL) was measured using quantitative polymerase chain reaction in 684 participants of the prospective population-based Bruneck Study (1995 baseline), with repeat RTL measurements performed in 2005 (n = 558) and 2010 (n = 479). Hazard ratios for T2DM were calculated across quartiles of baseline RTL using Cox regression models adjusted for age, sex, body-mass index, smoking, socio-economic status, physical activity, alcohol consumption, high-density lipoprotein cholesterol, log high-sensitivity C-reactive protein, and waist-hip ratio. Separate analyses corrected hazard ratios for within-person variability using multivariate regression calibration of repeated measurements. To contextualise findings, we systematically sought PubMed, Web of Science and EMBASE for relevant articles and pooled results using random-effects meta-analysis.

**Results:**

Over 15 years of follow-up, 44 out of 606 participants free of diabetes at baseline developed incident T2DM. The adjusted hazard ratio for T2DM comparing the bottom vs. the top quartile of baseline RTL (i.e. shortest vs. longest) was 2.00 (95% confidence interval: 0.90 to 4.49; P = 0.091), and 2.31 comparing the bottom quartile vs. the remainder (1.21 to 4.41; P = 0.011). The corresponding hazard ratios corrected for within-person RTL variability were 3.22 (1.27 to 8.14; P = 0.014) and 2.86 (1.45 to 5.65; P = 0.003). In a random-effects meta-analysis of three prospective cohort studies involving 6,991 participants and 2,011 incident T2DM events, the pooled relative risk was 1.31 (1.07 to 1.60; P = 0.010; *I* ^2^ = 69%).

**Conclusions/Interpretation:**

Low RTL is independently associated with the risk of incident T2DM. To avoid regression dilution biases in observed associations of RTL with disease risk, future studies should implement methods correcting for within-person variability in RTL. The causal role of short telomeres in T2DM development remains to be determined.

## Introduction

Telomeres are the extreme ends of eukaryotic chromosomes, stabilising and protecting the chromosome from degradation [1], [2]. Telomeres shorten with each cellular division, accelerated by inflammation and oxidative stress, and, below a critical length, the apoptotic programme of the cell is induced [1], [3].

Short telomeres are associated with increased risk of several age-related diseases [2] such as cardiovascular diseases [4]–[8] and cancer [9]–[11]. It has been suggested that biological ageing reflected by telomere length is also related to the development of type 2 diabetes (T2DM) [12]. Proposed pathophysiological mechanisms include a reduction in beta-cell mass [13], impaired insulin secretion [14] and adipocyte insulin resistance elicited by cellular senescence [15]. On the other hand, diabetic patients suffer from elevated levels of oxidative stress [16], which in turn leads to telomere attrition [17]. To disentangle these two effects and establish a temporal relationship, it is crucial to investigate the association of telomere length with risk of new-onset T2DM in studies with a prospective design. In such studies, telomere length is assessed before the diabetes diagnosis (although subclinical pre-diabetic changes may already be present). Furthermore, because telomere length fluctuates considerably over time [18]–[27], studies are needed that take into account the within-person variability of telomere length. Such analyses use repeated measurements instead of single baseline measurements to predict “long-term average” telomere length and therefore help estimate the “true” aetiological association with T2DM risk.

Our aims were three-fold. First, to assess the association of baseline leucocyte relative telomere length (RTL) with new-onset T2DM in the prospective population-based Bruneck Study. Second, to correct association estimates for long-term within-person variability of RTL. Third, to contextualise findings in a literature-based meta-analysis of all prospective evidence available to date.

## Material and Methods

### The Bruneck Study

The Bruneck Study is a prospective, population-based survey conducted in 1,000 individuals aged 40 to 79 years (125 per sex and decade of life). In 1990, participants were randomly selected from the inhabitants of the town of Bruneck (South Tyrol, Italy) and were examined every five years between 1990 and 2010 [5], [10], [19], [28], [29]. Participation rates exceeded 90% at all surveys. In the present study, we used leucocyte RTL measurements available from the surveys in 1995, 2005 and 2010 (n = 684, 558, and 479, respectively). Full medical records were available for review for all individuals, including those who did not participate in or died during follow up (100% follow up for clinical endpoints). The study protocol was approved by the local ethic committee of Bolzano (‘Comitato etico del comprensorio sanitario di Bolzano’; approval number 28–2010). All participants gave informed written informed consent before taking part and the study complies with the Declaration of Helsinki.

### Clinical history and examination at baseline

We assessed risk factors by validated standard procedures as previously described [5], [28]. Waist and hip circumferences were assessed with a plastic tape measure at the levels of the umbilicus and the greater trochanters respectively. We defined socioeconomic status on a three-category scale (low, medium or high) on the basis of information about occupational status and educational level of the person with the highest income in the household. High socioeconomic status was assumed if the participant had ≥12 years of education or an occupation with an average monthly income ≥$2,000 (baseline salary before tax). Low socioeconomic status was defined by ≤8 years of education or an average monthly income ≤$1,000. Physical activity was assessed using the validated Baeke Score [30]. Oxidation-specific biomarkers and other laboratory parameters were measured as previously described [31]. HbA1c was quantified using high performance liquid chromatography (DCCT-aligned assay). We estimated the degree of insulin resistance by homeostasis model assessment (HOMA-IR) using the formula fasting plasma glucose in mmol/l × fasting serum insulin in mU/l divided by 22.5 [32], with higher HOMA-IR values indicating higher insulin resistance. LDL cholesterol was estimated using the Friedewald equation [33].

### Measurement of leucocyte telomere length

Genomic DNA from the Bruneck Study blood samples was extracted from frozen EDTA-blood samples with the Invisorb Blood Universal Kit. DNA concentrations were measured with the Tecan NanoQuant infinite M200. Samples were normalised in 96-well microtiter plates. Leucocyte RTL was measured in a single laboratory by a single person within a short time period using a quantitative polymerase chain reaction (qPCR) approach developed by Cawthon [34] to measure T/S-ratios. The protocol was modified with regard to control samples and data processing as recently described [35]. T/S-ratios are proportional to individual telomere length. The composition of singleplex PCRs for telomere (T) and housekeeping gene (S) PCRs was identical. DNA samples were run in 15 µl reactions containing 1× Quantifast TM SYBR Green PCR mastermix (Qiagen), 10 ng of DNA, 1 µM of telomere primer or 250 nm of housekeeping gene 36B4 primer. The primer sequences (5′→3′) were: tel1b CGGTTTGTTTGGGTTTGGGTTTGGGTTTGGGTTTGGGTT; tel2b GGCTTGCCTTACCCTTACCCTTACCCTTACCCTTACCCT; 36B4u CAGCAAGTGGGAAGGTGTAATCC; 36B4d CCCATTCTATCATCAACGGGTACAA [5]. Each qPCR was carried out in 384-well format which was vertically segmented in two parts: one for the telomeres (T) and one for the housekeeping gene 36B4 (S). Each qPCR plate contained the standard DNA, a quality control (commercially available DNA-Human Genomic DNA, Roche), and a non-template control. All samples, standards, and controls were analysed in quadruplicate. All sample transfers and dilution steps were performed with a Tecan robotic workstation. Relative qPCR was carried out on an Applied Biosystems Taqman Fast Real-Time PCR 7900HT System. The thermal cycling began with the initial polymerase activation step (10 min at 95°C) and was followed by 40 cycles of 95°C for 15 s, 60°C for 1 min. The relative quantities were determined by the efficiency correction method [36], which does not require calibration curves and includes the individual real-time PCR efficiencies. Efficiencies were computed for all replicates of each sample. To check PCR data for outliers, the coefficients of variation (intra-assay CVs) were assessed for the Ct-values and the efficiency-values of quadruplicates in each gene. All single outlying values (CV>5%) were removed from further analyses. Further details were already published before [35]. Although RTL of samples from 1995 and 2005 was already measured before [5], [10], we reanalysed all samples of all time points together with the newly extracted samples from 2010 to avoid a measurement error over the time period. In addition, RTL measurement of samples of the same participant of all available time points were positioned on the same 384-well qPCR plate. After each qPCR run a melting curve analysis was performed to verify the specificity and identity of the products. The inter-assay coefficient of variation of T/S-ratios of the quality control, which was positioned on each 384-well plate (a total of 37 plates), was 11.8%. The laboratory personnel who performed the RTL measurements were blinded to the participants' status and outcome.

### Definition of type 2 diabetes mellitus

We established the diagnosis of T2DM according to the 1997 American Diabetes Association criteria (fasting glucose ≥126 mg/dl, i.e. ≥7 mmol/l) or when the subjects had a clinical diagnosis of T2DM and received anti-diabetic treatment [37]. Impaired fasting glucose was defined as a fasting glucose level of 100 to <126 mg/dl (i.e. 5.6 to <7.0 mmol/l). Self-reported T2DM status was confirmed by reviewing the medical records of the subject's general practitioners and Bruneck Hospital. A major strength of the Bruneck Study is that virtually all participants living in the survey area are referred to the same hospital and the network existing between hospital and practitioners allows retrieval of full medical information. There were no cases of type 1 diabetes mellitus in this cohort.

### Literature-based meta-analysis

Prospective studies of the association of telomere length with the risk of incident T2DM were sought using the databases PubMed, Web of Science and EMBASE. The search strategy combined keywords related to the exposure of interest (“telomere” or “telomeres”), the outcome (“diabetes”) and the study design (“cohort” or “prospective” or “longitudinal” or “hazard” or “risk” or “odds”). We included articles published before March 26^th^ 2014 and applied no language restrictions. We scanned the reference lists of identified studies and reviews for any additional relevant articles. Because of their vulnerability to reverse causation biases, retrospective case-control studies were excluded from the meta-analysis (whereas nested case-control studies conducted within prospective cohort studies were included). Study level characteristics and participant characteristics were extracted using a standardised data extraction form, including information on: geographic location, population source, year of baseline survey, number of participants, mean age and age range, percentage of males, telomere length assay method, number of incident T2DM cases, duration of follow-up, and reported measures of association (i.e. hazard ratios, odds ratios, or other measures of relative risk) with corresponding confidence intervals and degree of adjustment for confounders. If studies had reported measures of association for different degree of adjustment, the most adjusted estimate was used in the meta-analysis. We assessed the quality of the included studies with the Newcastle-Ottawa scale, a quality score ranging from zero to nine points [38].

### Statistical analysis

The statistical analysis was conducted according to a pre-specified analysis plan. Continuous variables were summarised as means (standard deviations) or medians (interquartile ranges), and dichotomous variables as numbers (percentages). Cross-sectional associations between RTL and other participant characteristics were determined using unadjusted and age- and sex-adjusted linear regression. To quantify within-person variability of RTL, regression dilution ratios were calculated using information from all available RTL measurements in 1995, 2005 and 2010 [39]. Regression dilution ratios can range from 0 to 1, with 1 indicating absence of within-person variability. Long-term average RTL was estimated by multivariate linear-mixed regression calibration model that allowed for a random intercept at the participant level [40].

The time-to-event analysis excluded participants with baseline T2DM. Person-years of follow-up were accrued from the baseline in 1995 until diagnosis of T2DM, death or October 1, 2010, whichever came first. Cox proportional hazard models were used to assess the association between RTL and T2DM incidence. Following a previous report of a possible threshold effect in associations of RTL with T2DM risk [41], we categorised study participants into groups of RTL quartiles and compared T2DM risk across these groups. The analyses were progressively adjusted for age, sex, body mass index, smoking, socio-economic status, physical activity, alcohol consumption, high-density lipoprotein cholesterol, log high-sensitivity C-reactive protein, and waist-hip ratio. The proportional hazards assumption was tested and confirmed using Schoenfeld residuals. To assess effect modification by sex, subsidiary analyses used interaction term between sex and RTL quartiles and tested for interaction with a likelihood ratio test. For the literature-based meta-analysis, we did not need to rescale published relative risks (RRs), because all eligible studies reported RRs on the same scale, i.e. for a comparison of extreme RTL quartiles. Hazard ratios (HRs) and odds ratios were assumed to approximate the same measure of RR. Reported RRs were pooled using random-effects meta-analysis. Heterogeneity between studies was quantified using the *I* ^2^ statistic and tested by a standard χ^2^ test [42]. Potential publication and small-study bias was formally assessed using Egger's test [43]. All analyses were conducted with Stata 12.0. A two-sided P value ≤0.05 was considered statistically significant. The presentation of results follows the recommendations by the STROBE and PRISMA guidelines (see **Checklists S1** and **S2**).

## Results

### Baseline characteristics

**Figure 1:**
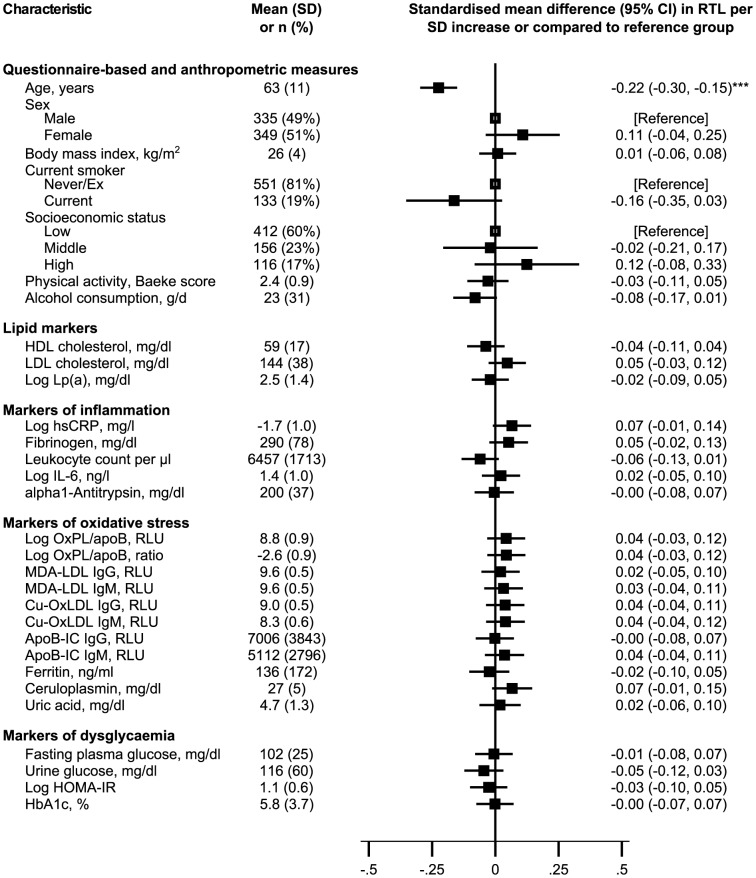
Baseline characteristics of the Bruneck Study population and their cross-sectional association with leucocyte relative telomere length (1995, n = 684). Standardised mean differences in leucocyte relative telomere length were adjusted for age and sex. Asterisks indicate level of statistical significance: *P≤0.05; **P≤0.01; ***P≤0.001. The mean (SD) of HbA1c was 5.8% (3.7%) in DCCT-derived units and 40 mmol/mol (17 mmol/mol) in SI units. Abbreviations: ApoB, apolipoprotein B; ApoB-IC, apoB-immune complexes; CI, confidence interval; Cu-OxLDL, copper-oxidised low-density lipoprotein; HbA1c, glycated haemoglobin; HDL, high-density lipoprotein; HOMA-IR, homeostatic model assessment of insulin resistance; hsCRP, high-sensitivity C-reactive protein; IgG, immunoglobulin G; IgM, immunoglobulin M; LDL, low-density lipoprotein; RTL, relative telomere length; MDA, malondialdehyde; OxPL/apoB, oxidised phospholipids on apolipoprotein B-100; SD, standard deviation; RLU, relative light unit; SMD, standardised mean difference; WHR, waist-hip ratio.

The median RTL at baseline was 1.05 T/S ratios (interquartile range: 0.92–1.21). **Figure 1** summarises baseline characteristics of the study population (n = 684). The mean age of study participants was 63 years (SD, 11) and 49% were men. The multivariable adjusted regression dilution ratio of RTL was 0.68 (95% confidence interval: 0.61 to 0.76). We investigated the age- and sex-adjusted cross-sectional association at baseline between several characteristics and standardised RTL (**Figure 1**). The strongest association was observed with age. On average, every one standard deviation older age (11 years) was associated with 0.22 shorter standardised RTL (−0.30 to −0.15; P = 2×10^−9^). There was no significant correlation of RTL with other parameters, including markers of inflammation, oxidative stress and hyperglycaemia (all P>0.05).

**Figure 2:**
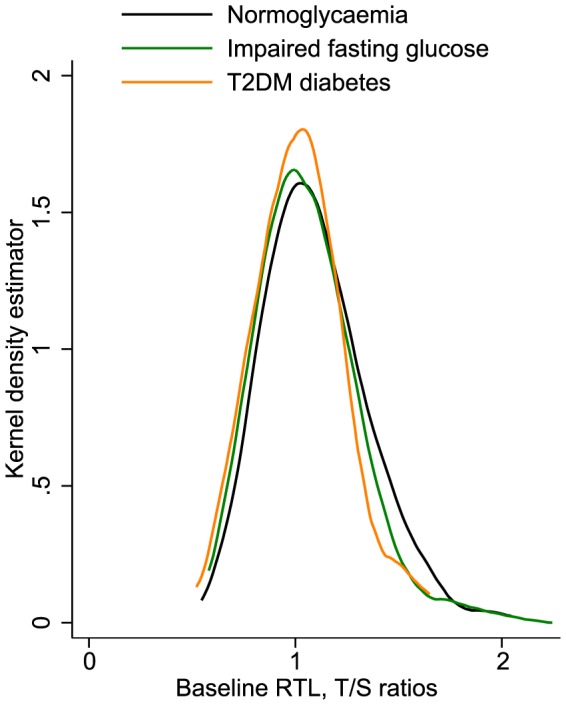
Distribution of baseline leucocyte relative telomere length in the Bruneck Study according to different disease states (1995, n = 684). Abbreviations: RTL, relative telomere length; T2DM, type 2 diabetes mellitus. There were 390 participants with normoglycaemia, 216 participants with impaired fasting glucose, and 78 participants with a clinical diagnosis of T2DM.

We further investigated whether baseline RTL differed according to baseline T2DM status (**Figure 2**). We observed no significant difference in RTL across the groups of normoglycaemic participants (n = 390), participants with impaired fasting glucose levels (n = 216), and participants with a clinical diagnosis of T2DM (n = 78) (P_trend_ = 0.346).

### Association of leucocyte telomere length with risk of incident type 2 diabetes mellitus

**Figure 3:**
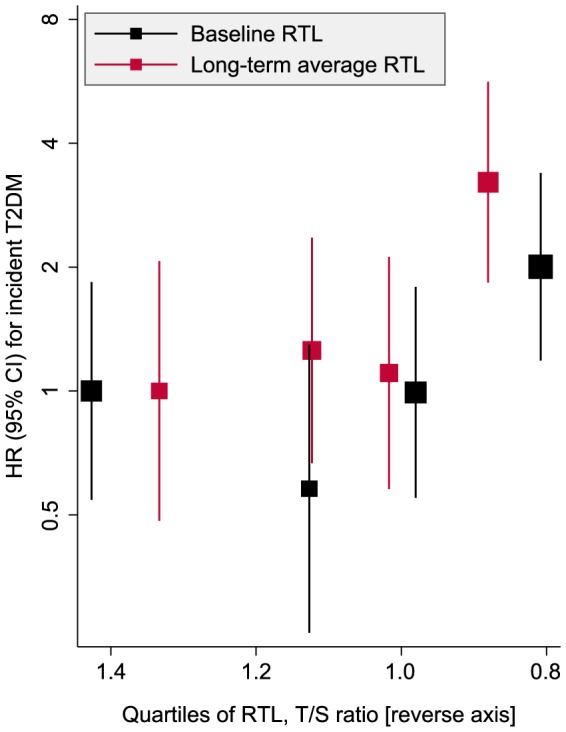
Association of leucocyte relative telomere length an risk of type 2 diabetes mellitus in the Bruneck Study (n = 606, 44 events over follow-up 1995–2010). Cox models were adjusted for age, sex, body mass index, smoking, socio-economic status, physical activity, alcohol consumption, high density lipoprotein cholesterol, log high-sensitivity C-reactive protein, and waist-hip ratio. Abbreviations: CI, confidence interval; HR, hazard ratio; RTL, relative telomere length; T2DM, type 2 diabetes mellitus.

The Cox regression analyses excluded 78 participants with a baseline diagnosis of T2DM. Between 1995 and 2010, 44 of the 606 individuals in the study population developed T2DM (incidence rate, 5.8 per 1,000 person years [4.3 to 7.8]). **Table 1** compares T2DM risk across quartiles of decreasing RTL. In a comparison of the bottom vs. top quartile of baseline RTL, the age- and sex-adjusted HR was 1.89 (0.85 to 4.18; P = 0.116). The most adjusted model yielded a HR of 2.00 (0.90 to 4.49; P = 0.091). In a comparison of the bottom quartile of baseline RTL vs. the remainder, the respective HRs were 2.21 (1.17 to 4.16; P = 0.015) and 2.31 (1.21 to 4.41; P = 0.011). **Table 1** also shows analyses using long-term average RTL. In the most adjusted model, participants in the bottom quartile of long-term average RTL had a HR of 3.22 (1.27, to 8.14; P = 0.014) compared with the top RTL quartile and 2.86 (1.45 to 5.65; P = 0.002) compared with all other quartiles. We observed that increases in T2DM risk were confined to the bottom RTL quartile (particularly when using long-term average RTL), in line with a possible threshold effect (**Figure 3**). Results were similar in analyses that excluded the first five years of follow-up (data not shown). There was no evidence for differential associations in women and men (likelihood-ratio tests for interaction: P = 0.776 for baseline RTL; P = 0.571 for long-term average RTL).

**Table 1:** Association of leucocyte relative telomere length with incident type 2 diabetes mellitus in the Bruneck Study (n = 606, 44 events).

Exposure/adjustment	Quartile 1	Quartile 2	Quartile 3	Quartile 4	Quartile 4 vs. remainder
	(Longest RTL)			(Shortest RTL)	
**Baseline RTL**					
Median (range) of RTL, T/S ratios	1.35 (1.21–3.87)	1.13 (1.05–1.21)	0.98 (0.92–1.05)	0.82 (0.52–0.91)	
Incidence rate of T2DM per 1,000 person-years	5.2 (2.9, 9.4)	3.1 (1.4, 6.9)	5.4 (3.0, 9.8)	10.6 (6.5, 17.2)	
Hazard ratio for T2DM (95% CI)					
Adjusted for age and sex	1.00 [Reference]	0.58 (0.22, 1.58)	0.95 (0.41, 2.22)	1.89 (0.85, 4.18)	2.21 (1.17, 4.16)*
Plus BMI, smoking, SES, and physical activity	1.00 [Reference]	0.57 (0.21, 1.56)	0.96 (0.41, 2.24)	1.89 (0.85, 4.21)	2.22 (1.17, 4.21)*
Plus alcohol consumption, HDL-C, log hsCRP, and WHR	1.00 [Reference]	0.58 (0.21, 1.58)	0.99 (0.42, 2.32)	2.00 (0.90, 4.49)	2.31 (1.21, 4.41)*
**Long-term average RTL**					
Median (range) of RTL, T/S ratios	1.29 (1.18–2.94)	1.12 (1.06–1.18)	1.02 (0.97–1.06)	0.89 (0.63–0.96)	
Incidence rate of T2DM per 1,000 person-years	3.6 (1.8, 7.3)	5.0 (2.7, 9.4)	4.5 (2.3, 8.6)	12.2 (7.6, 19.6)	
Hazard ratio for T2DM (95% CI)					
Adjusted for age and sex	1.00 [Reference]	1.36 (0.53, 3.45)	1.16 (0.44, 3.05)	3.24 (1.29, 8.15)*	2.76 (1.41, 5.41)**
Plus BMI, smoking, SES, and physical activity	1.00 [Reference]	1.24 (0.48, 3.20)	1.10 (0.41, 2.92)	3.05 (1.21, 7.70)*	2.73 (1.39, 5.36)**
Plus alcohol consumption, HDL-C, log hsCRP, and WHR	1.00 [Reference]	1.25 (0.49, 3.23)	1.11 (0.42, 2.94)	3.22 (1.27, 8.14)*	2.86 (1.45, 5.65)**

Asterisks indicate level of statistical significance: *P≤0.05; **P≤0.01; ***P≤0.001. Abbreviations: BMI, body mass index; HDL-C, high density lipoprotein cholesterol; hsCRP; high-sensitivity C-reactive protein; RTL, relative telomere length; SES, socio-economic status; T2DM, type 2 diabetes mellitus; WHR, waist-hip ratio. Participants with a baseline history of type 2 diabetes mellitus were excluded from the analysis (n = 78).

Subsidiary analysis evaluated the association of a baseline diagnosis of T2DM with RTL change over 15 years. There was no significant difference in RTL dynamics according to baseline T2DM status (mean standardised RTL change in participants with vs. without T2DM at baseline [95% confidence interval]: −0.068 [−0.450 to 0.314]; P = 0.727). This analysis had limited power because it included only 400 study participants with RTL measurements available both in 1995 and 2010, of which only 20 had T2DM at baseline.

### Literature-based meta-analysis

**Figure 4:**
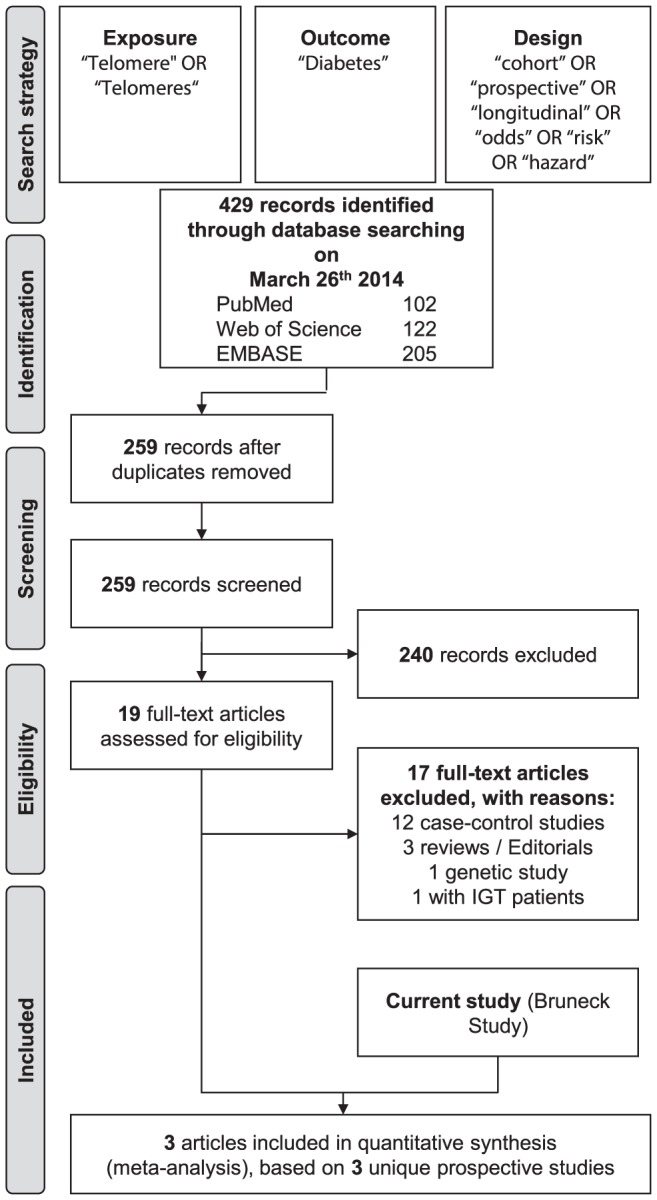
Study flow diagram of the literature-based meta-analysis. The figure is based on the 2009 PRISMA flow diagram template (available from http://www.prisma-statement.org/statement.htm).

Our literature search retrieved 429 records (**Figure 4**). We excluded 170 duplicate records and 240 records on the basis of their title and abstract. We carefully checked the full-text of the remaining 19 records and excluded a further 17 articles, including 12 case-control studies and 1 study that selectively recruited participants with impaired glucose tolerance [44]. Finally, together with the Bruneck Study, we identified three relevant prospective studies eligible for inclusion in the literature-based meta-analysis [41], [45].

**Figure 5:**
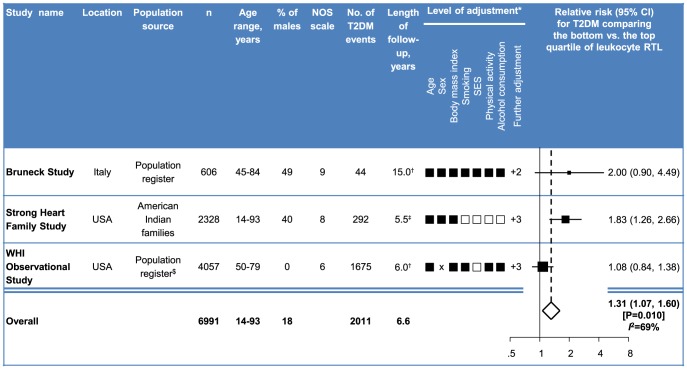
Description and meta-analysis of published data from three prospective cohort studies on the association of short telomeres and risk of type 2 diabetes mellitus. Published relative risks were pooled by random-effects meta-analysis. In the Bruneck Study and the Strong Heart Family Study, type 2 diabetes was defined according to the 1997 American Diabetes Association criteria. In the WHI Observational Study, diabetes was defined based on self-report and hospitalisation for type 2 diabetes. All three studies measured telomere length with a quantitative polymerase chain reaction technique. *Reported relative risks were additionally adjusted for two variables in the Bruneck Study (HDL cholesterol and log hsCRP), three variables in the Strong Heart Family Study (age^2^, fasting glucose, total triglycerides), and three variables in the Women's Health Initiative Observational Study (date of blood collection, clinical centre, hormone therapy). †Max. ‡Mean. $The Women's Health Initiative Observational Study involved postmenopausal women who proved to be ineligible or unwilling to be randomised as part of the Women's Health Initiative Clinical Trial. Abbreviations: CI, confidence interval; NOS, Newcastle-Ottawa Scale for assessing the quality of nonrandomised studies in meta-analyses; T2DM, type 2 diabetes mellitus; WHI, Women's Health Initiative.

The characteristics of the three studies are summarised in **Figure 5**. The Strong Heart Family Study is a multigenerational study of American Indian families in four states of the USA, which recorded 292 incident T2DM events over a mean of 5.5 years (defined according to the 1997 American Diabetes Association criteria) [41]. The observational arm of the Women's Health Initiative used a nested case-control analysis that included 1,675 incident cases of T2DM (defined based on self-report or T2DM hospitalisation) [45]. Overall, information from the three studies was available on 6,991 participants and 2,011 incident T2DM events recorded over a weighted mean of 6.6 years. Baseline age of participants ranged from 14 to 93 years; 18% were male. The reported multivariable adjusted RRs for T2DM were quantitatively similar in the Bruneck Study and the Strong Heart Family Study (**Figure 5**). The pooled RR for all three studies was 1.31 (1.07 to 1.60; P = 0.010) for a comparison of the bottom vs. the top quartile of baseline RTL. Between-study heterogeneity was moderate with an *I* ^2^ of 69% (0% to 91%) (**Figure 5**). There was no evidence of publication bias (P = 0.460). We could not calculate a pooled RR corrected for long-term variability, because such an estimate was only available for the Bruneck Study.

## Discussion

In the present report, we demonstrate a significant positive association between shorter leucocyte telomeres and T2DM risk. In the Bruneck Study, the increase in risk appeared to be confined to the bottom quartile of RTL (i.e. participants with the shortest RTL) and was particularly evident in analyses correcting for long-term variability in RTL. In a pooled analysis of published reports from three prospective cohort studies, we estimated that people in the bottom RTL quartile had a 31% (7 to 60%) higher risk of developing T2DM than those in the longest RTL quartile.

RTL is a dynamic measure that may decrease but also increase over time [18]–[27]. Possible sources of variability in measured RTL include the (i) cumulative effect of environmental and behavioural exposures, (ii) varying telomerase activity, (iii) stress-induced repopulation of peripheral blood by recently dividing hematopoietic bone marrow cells, (iv) shifts in the cellular composition of peripheral blood leucocytes (differential blood count) and release of leucocyte subpopulations during acute infections [46], [47] (i.e. “true variation”), and (v) measurement error. Previous epidemiological studies on RTL and T2DM risk have not been able to correct for RTL variability, potentially yielding biased estimates. The Bruneck study, which made such a correction on the basis of up to three repeated measurements taken over a follow-up time of 15 years, indicates an independent association of shorter telomeres with T2DM, of greater magnitude than previously reported. The within-person variability of RTL was comparable to that of commonly measured cardio-metabolic risk markers, such as blood pressure or high-sensitivity C-reactive protein. It was lower than previously reported [19] because we further optimised our technique for RTL quantification, including consistent DNA extraction method and re-measurement in quadruplicate.

Previous studies have suggested a possible threshold effect of associations of RTL with age-related diseases, including T2DM [41]. Our study independently confirms this hypothesis, demonstrating an elevation in T2DM risk only in the quartile of participants with the shortest telomeres. As suggested by Zhao *et al*. [41], one intriguing biological explanation for this finding could be the crossing of the “Hayflick limit” [3], beyond which cells cease to divide and undergo apoptosis. Furthermore, several pieces of evidence support that short telomeres play an important role in T2DM pathogenesis, rather than being an epiphenomenon of a pre-diabetic metabolic state. In mice, deletion of the telomerase RNA component (TERC) lowers the replication capacity of beta-cells and thereby leads to a reduced islet mass and failure to produce adequate amounts of insulin in response to glucose stimulation and high fat diet [13]. It has also been proposed that short telomeres impede insulin secretion through inhibition of calcium-mediated exocytosis [14]. In adipocytes, cellular senescence leads to insulin resistance, which is reversible by inhibition of p53 activity [15]. In a study of 22,715 women and 1,445 incident T2DM events, Zee *et al*. have shown that genetic variants related to telomere pathways are associated with T2DM risk [48]. On the other hand, evidence suggests that diabetes has a marked impact on telomere length dynamics. High levels of oxidative stress observed in diabetes patients [16] accelerate telomere attrition because the high guanine content of telomeres makes them particularly vulnerable to reactive oxygen species [17]. Xu *et al.* demonstrated that new-borns have significantly shorter telomeres if their mother suffered from gestational diabetes [49]. However, the Nurses' Health Study has quantified genetic predisposition to T2DM with a risk score combining 36 genetic variants and ruled out a strong causal impact of T2DM on telomere dynamics (although the study was powered to only detect effects of ≥9% explained variability) [50].

To place our findings in context of the currently available epidemiological literature, we performed a systematic literature review and meta-analysis of the available published evidence from prospective cohort studies on the topic. The pooled RR for a comparison of bottom vs. top quartiles of telomere length (i.e. shortest vs. longest) involving data from 6,991 participants and 2,011 incident T2DM cases was 1.31 (1.07 to 1.60). In contrast to an earlier meta-analysis [12], we excluded articles reporting on case-control studies, thereby limiting the effect of reverse causation biases in our analysis. However, because the present evaluation was based on observational studies, we cannot fully exclude the possibility that our estimates are confounded by other factors or the consequence of the often extended lag time between manifestation and diagnosis of T2DM.

Our study has several strengths. First, the Bruneck Study is representative of the general population (with a recruitment process using municipal registers and with a response rate>90%). It therefore crucially expands our knowledge from select populations (i.e. populations from specific ethnic backgrounds or other non-representative samples) to the healthy general community. Second, the Bruneck cohort is extremely well-characterised with 100% follow-up over 15 years and high-quality ascertainment of both clinical endpoints and potential confounders. The detailed characterisation of the study participants helped us estimate independent associations adjusted for a large panel of proposed risk markers for T2DM, including adiposity measures, smoking, social class, and physical activity. Third, the present study had a prospective design and used rigorous baseline examinations to exclude all individuals with T2DM at baseline, thereby minimising any reverse causation biases. Supplementary analyses that excluded the first five years of follow-up yielded qualitatively similar results. Fourth, to enhance validity of the measurement, we extracted all available DNA samples with the same DNA extraction kit. Although samples taken in 1995 and 2005 had been previously analysed [5], [10], samples from all three time points were reanalysed simultaneously to avoid a measurement error over the time period. In addition, a bias according to the ascertainment of RTL on different qPCR plates was avoided by positioning the available DNA samples from a single individual from different time points side by side on the same plate. To maximise accuracy, RTL measurements were performed in quadruplicate, in an intensively standardised and automated manner, and within a short period of time by personnel blinded to the characteristics and outcome of the study participants. We have demonstrated previously that RTL measurement using qPCR, as performed in the Bruneck Study, is highly correlated with measurement of absolute telomere length using a Southern Blot technique (r = 0.765) [5]. Fifth, we were able to model long-term average RTL and provide effect estimates for T2DM corrected for fluctuations in RTL over time. Finally, we performed a comprehensive literature review and combined data from previously published prospective studies.

Our study also has potential limitations. First, the Bruneck Study population was entirely Caucasian and therefore findings are only generalizable to this ethnicity. Previous investigations by the Nurses' Health Study on differences by ethnicity (Caucasian, African Americans, Hispanic, and Asian) have shown generally comparable associations of RTL with T2DM incidence [45]. Second, telomere length was measured in circulating blood leucocytes only. More precise assessments and comparisons of telomere length in different tissues (e.g. liver, muscle, adipose tissue, and pancreas) would be helpful to better understand the role of telomeres in disease development but, clearly, this is not feasible in large population studies. Third, the number of incident T2DM events was low in the Bruneck Study limiting our ability to conduct more detailed investigations (e.g. extensive subgroup analyses), although major findings were independently confirmed in the literature-based meta-analysis. Fourth, the meta-analyses of published data used single baseline measurements of RTL to study the association with subsequent T2DM. Our analysis of RTL reproducibility over up to 15 years suggests that previous studies could have substantially underestimated potential associations with T2DM. Finally, because the present evidence is limited to observational studies in primarily adult populations, no judgement on the causal involvement of telomere length in T2DM can be made on the basis of the present report.

## Conclusion

Low RTL is independently associated with the risk of incident T2DM. To avoid regression dilution bias in observed associations of RTL with disease risk, future studies should implement methods correcting for within-person variability in RTL. Whether there is a causal involvement of telomeres in T2DM development remains to be determined.

## Supporting Information

Checklist S1 **STROBE Statement—Checklist of items that should be included in reports of cohort studies.**
(PDF)

Checklist S2 **PRISMA 2009 Checklist.**
(PDF)

File S1 **Underlying data for** **Figure 1** **,** **3** **and** **5** **.**
(XLSX)
